# Polymer@gold Nanoparticles Prepared via RAFT Polymerization for Opto-Biodetection

**DOI:** 10.3390/polym10020189

**Published:** 2018-02-14

**Authors:** Sónia O. Pereira, Ana Barros-Timmons, Tito Trindade

**Affiliations:** Department of Chemistry, CICECO-Aveiro Institute of Materials, University of Aveiro, 3810-193 Aveiro, Portugal; anabarros@ua.pt (A.B.-T.); tito@ua.pt (T.T.)

**Keywords:** RAFT, nanocomposites, gold nanoparticles, plasmonic nanostructures, bioapplications

## Abstract

Colloidal gold nanoparticles (Au NPs) have been used in several biological applications, which include the exploitation of size- and shape-dependent Localized Surface Plasmon Resonance (LSPR) in biosensing devices. In order to obtain functional and stable Au NPs in a physiological medium, surface modification and functionalization are crucial steps in these endeavors. Reversible addition-fragmentation chain transfer (RAFT) polymerization meets this need offering the possibility of control over the composition and architecture of polymeric shells coating Au NPs. Furthermore, playing with a careful choice of monomers, RAFT polymerization allows the possibility to design a polymer shell with the desired functional groups aiming at Au based nanocomposites suitable for biorecognition and biotargeting. This review provides important aspects concerning the synthesis and optical properties of Au NPs as well as concepts of RAFT polymerization. Understanding these concepts is crucial to appreciate the chemical strategies available towards RAFT-polymer coated Au core-shell nanostructures, which are here reviewed. Finally, examples of applications in opto-biodetection devices are provided and the potential of responsive “smart” nanomaterials based on such structures can be applied to other biological applications.

## 1. Introduction

**Figure 1:**
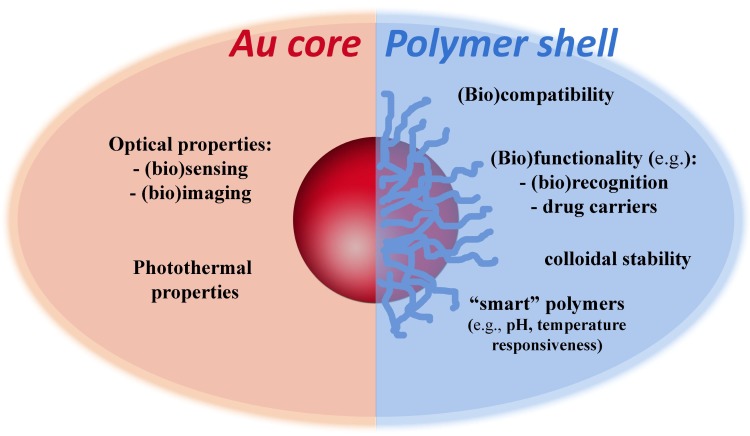
Functionalities associated to polymer/Au nanocomposite particles in some bioapplications. Inspired in [1].

The optical properties of colloidal gold nanoparticles (NPs) have been widely explored in diverse applications and in certain cases associated to polymer coatings. Very often, such hybrid polymer/gold nanostructures comprise Au cores decorated with polymer shells. While the Au NPs are thought to provide optical response due to their plasmonic behavior, the resulting polymer coatings might confer robustness, stability, functionality, responsiveness and biocompatibility, to the final nanocomposite (Figure 1). These potential benefits demand judicious control over the morphological properties of the Au cores and also on their surface modification when using polymers. The latter can be achieved by a number of strategies that include living radical polymerization techniques such as RAFT (Reversible Addition-Fragmentation chain Transfer).

This review explores the plasmonic properties of Au NPs aiming at the development of sensing platforms for biodetection, focusing on the surface functionalization of Au NPs using polymers prepared via RAFT polymerization. Therefore, the first part of the review presents a brief overview of the synthesis and properties of colloidal Au NPs as well as of surface functionalization methods applied to such colloids. In the second part, the main concepts underlying RAFT polymerizations are presented and the approaches that have been used to prepare polymer/Au nanocomposites are discussed. The third part of this review concerns the use of RAFT to prepare polymer coated Au NPs as optical biosensors and eventual multifunctionality in theranostic methods applied for example in cancer therapy.

## 2. Colloidal Gold Nanoparticles: Optical Properties and Related Chemical Practices

**Figure 2:**
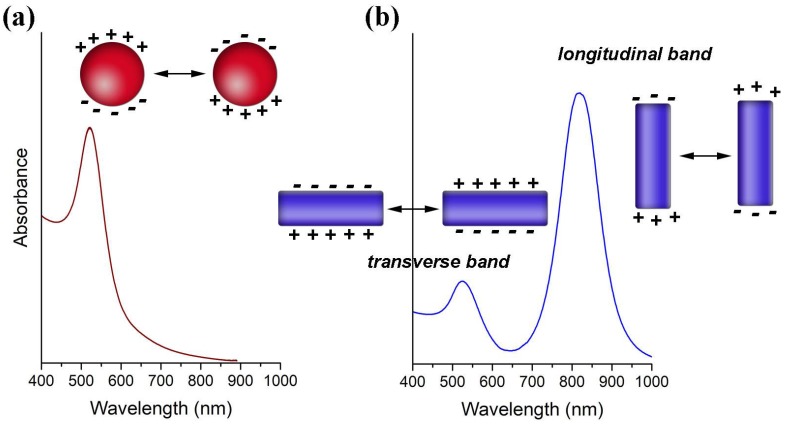
Schematic illustration of the free electron coherent oscillation at a given wavelength for the excitation radiation and corresponding optical spectra of Au nanospheres (**a**) and nanorods (**b**), evidencing the Localized Surface Plasmon Resonance (LSPR) bands.

Colloidal Au NPs have been used in several bioapplications mainly due to properties associated to the existence of a Localized Surface Plasmon Resonance (LSPR) [2,3,4,5]. The LSPR is a collective oscillation of the conduction electrons confined to the metal particle dimensions (Figure 2). As such, it occurs for particles much smaller than the wavelength of the incident light that in this situation probes the whole electron cloud. The interaction with the electromagnetic radiation originates accumulation of surface charge at opposite regions of the particle, that result in a restoring force due to oscillating dipoles whose electric field is opposed to the incident field. There is a characteristic resonance frequency for the oscillating dipoles originated by the polarization of the conduction electrons in the metal NPs. For small Au NPs (e.g., 15 nm), the LSPR frequency is located in the visible spectrum, giving rise to strong absorption when irradiated with light at that frequency. Light absorption and scattering at this wavelength region, which is stronger as compared to molecular dyes and becomes more relevant for bigger particles, explains the red color of such colloidal Au NPs.

**Figure 3:**
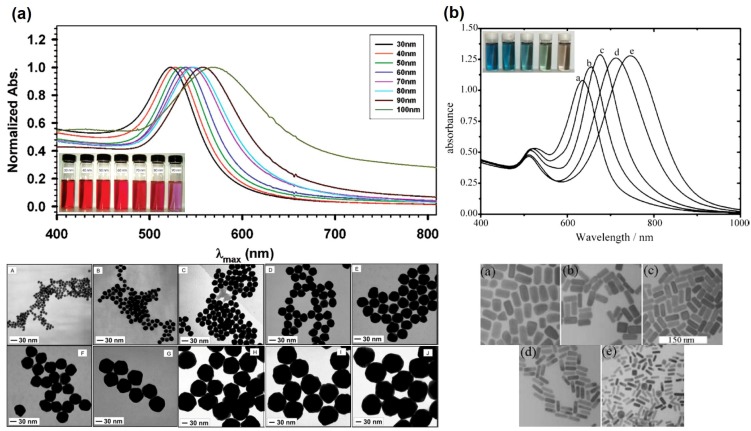
Absorbance spectra, photographs and TEM micrographs of gold nanospheres with different sizes (**a**) and gold nanorods with different aspect ratios (**b**). Adapted with permission from [7]. Copyright 2007 American Chemical Society; and adapted from [8] Copyright 2005 with permission from Elsevier, respectively.

The LSPR of Au NPs is influenced by their size and shape, by the dielectric constant of the surrounding medium and the distance between neighboring nanoparticles [2,6,7,8]. Although for Au nanospheres of bigger sizes and anisotropic particles such as Au nanorods (NRs), the explanation becomes more complex, it suffices to state here that the optical spectra show significative differences. Hence, by increasing the NPs diameter the LSPR shifts to higher wavelengths as shown in Figure 3a [7]. For Au NRs the differences in the optical spectra are even more pronounced, showing an absorption band (transverse mode) corresponding to the electron cloud oscillation along the short axis (diameter), and another band (longitudinal mode) corresponding to the electron cloud oscillation along the long axis (Figure 3b). For the latter, the wavelength for maximum absorption is particularly sensitive to the length of the NRs. Figure 3b shows that by increasing the aspect ratio (AR = length/width) of Au NRs, the longitudinal band shifts to higher wavelengths and can reach the near-infrared (NIR) region, which is of great interest for bioapplications [8,9].

The relevance of the LSPR of Au NPs for bioapplications is clearly perceived by taking in consideration that under light excitation at such frequencies, the systems will tend to relax either by scattering or by heat dissipation. The first process can be explored in optical biolabeling or biosensing for which a relatively diminutive amount of Au is required. The second process can be explored in photothermia namely by using Au NRs that show the longitudinal LSPR in the NIR therapeutic window (650–1350 nm), because excitation light of maximum penetration depth in biological tissues can be used. Additionally, the optical properties observed for Au colloids can be explored by associating these nanostructures to other optical active compounds, such as molecular fluorophores, which potentiate the use of Au NPs in several biosensing applications. Hence, the presence of certain fluorophores may experience fluorescence quenching when they are in the vicinity of AuNPs due to Fӧrster resonance energy transfer (FRET) as well as to Nanoparticle Surface Energy Transfer (NSET); the latter is observed at distances nearly twice as far as FRET [10,11,12,13,14]. In the FRET effect, the fluorescence emission band of the donor (fluorophore) overlaps the absorption band of the acceptor (Au NPs) resulting in several vibronic transitions in the donor that have practically the same energy as the corresponding transitions in the acceptor. The critical distance between donor and acceptor (e.g., using organic dyes) is up to 10 nm, for occurring energy transfer in conventional FRET. However, energy transfer between fluorophores and metal NPs has been described for larger distances due to the so called NSET. For distances between 2–30 nm an efficient energy transfer occurs between the dye and the NP, while for longer distances (>50 nm) the energy transfer can suffer oscillations [14,15,16,17,18].

There are several well-established methods to synthesize Au NPs, most of them are based on the reduction of a gold (III) salt in the presence of a stabilizing agent, whose synthesis conditions are varied in order to produce colloids characterized by distinct particle size distributions and stable either in aqueous or organic medium. The increasing interest in using Au NPs for a number of applications led to the development of new methods in which reducing agents have been used as more environmentally friendly colloidal stabilizers [19,20,21]. Some of the most relevant methods used to prepare Au NPs will be presented as follows.

The most popular method to synthesize Au NPs has been the citrate method, originally introduced by Turkevich et al. [22], and which involves the formation of Au colloids by the reduction of boiling tetrachloroauric acid (HAuCl_4_) using sodium citrate (Na_3_[(COO)_3_C_3_H_5_O]), in aqueous medium. The citrate method allows control over the average particle diameter within a wide range (~10–50 nm) varying the concentration ratio between the gold salt and sodium citrate. The citrate method yields NPs stabilized by interparticle electrostatic repulsions due to the adsorption of citrate anions at the particles’ surfaces, which are thus negatively charged [23,24]. Monodisperse and quasi-spherical Au NPs with average diameters up to 300 nm, have been reported via a seeded growth method using the citrate coated Au NPs as seeds [25,26].

Smaller Au NPs can be synthesized by the method reported by Brust and Schiffrin in 1994 [27], also known as the two-phase synthesis. In this method, the [AuCl_4_]^−^ precursor species are transferred from the aqueous solution to an organic solvent (e.g., toluene) via a phase-transfer reagent and then reduced with aqueous sodium borohydride, at room temperature, in the presence of a long chain thiol (e.g., dodecanethiol), thus yielding thiol capped Au NPs which are stable in organic medium. In this case, the average particle size is varied by varying the alkanethiol:HAuCl_4_ molar ratio from 0:1 (8 nm) to 2:1 (2 nm). Although the reaction mechanism is not well understood, an increase of the amount of alkanethiol results in a decrease of the NPs average size regardless the amount of reducing agent employed [28]. The methods of phase exchange are particularly relevant because aqueous stable Au NPs are normally required for bioapplications. Noteworthy, hydrophobic Au NPs can be transferred from organic to aqueous media using for example DMAP (4-(dimethylamino)pyridine), which acts as a stabilizer and transfer agent [29,30]. Methods using borohydride as a reducing agent in one-phase synthesis, namely in aqueous medium, have also been reported. In these procedures, thiol compounds are very often used as colloidal stabilizers and the particle size can be controlled by varying the ratio between the thiol ligand, the Au(III) species and the reducing agent [31,32]. Also, sodium citrate can be used in this case only as stabilizer since it does not reduce Au(III) extensively at room temperature [33]. However, there are reports on the synthesis of aqueous stable Au NPs using borohydride as a reducing agent without addition of further stabilizers during the synthesis but with judicious control on the reacting mixture pH, in this case set at 8 [34,35]. Indeed, depending on the pH of the reacting mixture, the hydrolysis of the initial precursor [AuCl_4_]^−^ results in Au(III) hydroxo complexes whose distinct chemical reactivity impacts on the nucleation and growth stages of the Au NPs, which in turn influences the final morphology observed for the NPs [36,37]. On the other hand, Yang et al. [38] have used both citrate and borohydride anions as reducing agents and have reported a major influence for the chloride concentration on the particle size distribution of Au NPs. The authors argued that by increasing the chloride concentration, the aggregation of the primary particles is promoted due to the decrease of surface charge, thereby leading to bigger particles. Hence, by controlling the pH and the chloride concentration, NPs with diameters in the 19–47 nm range were obtained by using sodium citrate as reducing agent and, Au NPs with diameters ranging 3–12 nm were obtained by using sodium borohydride as the reducing agent. Other authors even explored green synthesis methods to prepare nanoparticles, in this case using plant extracts as reducing and stabilizing agents [39].

The syntheses of Au NPs have not been limited to nearly spherical nanostructures and anisotropic NPs have been reported, such as rods, prisms, wires and stars. In fact, the exploitation of distinct optical properties of Au NPs of variable morphology has been a cornerstone on the development of chemical strategies to functional nanomaterials. In a number of synthesis methods, the oriented particle growth occurs from Au(0) seeds (*d* < 10 nm) previously introduced in a growth solution containing the Au(III) precursors, a soft reducing agent (e.g., ascorbic acid) and a shape-directing agent, such as an halide counter ion (Cl^−^, Br^−^ and I^−^) [40,41]. For example, NPs with a rod shape (nanorods, NRs) have been prepared using the seed-mediated growth method introduced by Murphy et al. 2001 [42] which was later improved, in order to obtain better control over the particle aspect ratio (AR) and thus allowing the synthesis of NRs with higher AR [43,44,45]. The first step of this method concerns the preparation of Au^0^ seeds (1.5–5 nm in diameter) using BH_4_^−^ as reducing agent and then mixing the ensuing seeds with a growth solution. The growth solution contains the Au(III) salt, ascorbic acid, the surfactant CTAB (hexadecyltrimethylammonium bromide) and, in some cases, AgNO_3_. The ascorbic acid is used as a mild reducing agent in order to promote the growth of the Au particles but avoiding extensive reduction that would cause further nucleation events that would result in a polydispersed colloid. The surfactant CTAB used as stabilizing agent is very important to control the AR, namely the length of the NR, because it is used as a soft template for shaping the nanoparticle into a rod but also because CTAB molecules adsorb preferentially onto the faces along the length of the NR ({110} or {100} faces), so the growth is promoted at the ends of the NR, {111} faces, and a slow growth of the NR width is observed. Additionally, the presence of chemisorbed Ag^+^ ions helps the shape induction. According to Murphy et al., Ag^+^ is adsorbed at the Au NPs surface in the form of AgBr (Br^−^ ions come from the CTAB) restricting the growth in that specific facet ({110}). It is noteworthy that Ag NPs are not generated in this case [42,43,44,45,46] and other parameters influence the growth of Au NRs using these methodologies [47]. Although controversial, the presence of bromide ions seems to be essential to control the synthesis of Au NRs. Nevertheless, the synthesis of monodispersed Au NRs using bromide-free surfactants has also been described [48].

**Figure 4:**
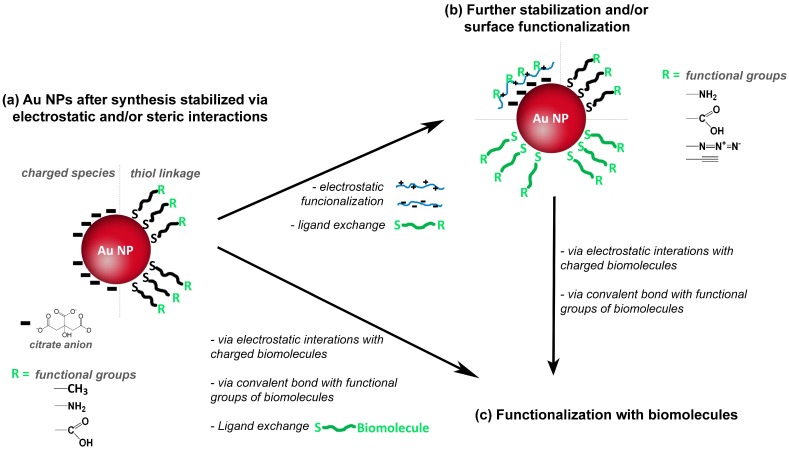
Schematic representation of surface stabilization and or functionalization of gold nanoparticles (Au NPs), followed by surface (bio)functionalization.

Although colloidal Au NPs have colloidal stabilizers at the surfaces, additional surface modification is very often necessary to ensure further functionalization envisaging specific applications [4,49]. The functionalization of NPs is of great interest because it mediates the interaction of the Au NPs with the surroundings, namely via surface charges and functional groups (e.g., carboxyl or amine groups), which allows for example the bio-labelling of Au NPs using antibodies or DNA strands. An example of the former strategy is the use of DMAP (4-(dimethylamino)pyridine), which can be attached to Au NPs for conferring positive surface charge [50]. Figure 4a illustrates the use of thiol compounds whose chemical affinity for Au is well known and is an example of the acid-base interplay between a soft Lewis acid and a soft Lewis base. Therefore, thiol ligands such as sodium 10-mercaptodecanesulfonate [51] or polymers containing thiol groups have been used to modify the surface of Au NPs. Others polymers such as charged polyelectrolytes can be used for surfaces modification via electrostatic interactions [51,52,53,54,55,56,57], see Figure 4b. Regarding the functionalization of the NPs using biomolecules (Figure 4c), two main strategies can be distinguished. One strategy involves electrostatic interactions between surface charged Au NP and charged biomolecules; the other strategy involves formation of covalent bonds between functional groups at the surface of Au NP and functional groups of the biomolecules. Alternatively, a biomolecule modified with a thiol group is directly used for ligand exchange providing, in one step, stability and functionalization; this strategy is commonly used for functionalization with DNA [58,59].

Among the several methods of surface modification and functionalization of Au NPs, those using polymers have received increasing interest in the last few years. This is because polymers offer the possibility to prepare stable, robust and multifunctional shells keeping the optical properties of the Au core, but also the polymeric shell can be responsive to an external stimulus, such as pH or temperature [60,61]. On the other hand, these endeavors require a better control on the polymerization techniques employed to coat the NPs surfaces, which makes RAFT a very attractive strategy.

## 3. RAFT Polymerization

### 3.1. General Concepts

Reversible-deactivation radical polymerization (RDRP), historically known as living or controlled radical polymerization (CRP) [62], allows the synthesis of block copolymers and complex polymer structures with narrow and controlled molecular weights [63,64,65,66,67]. The most known and widely used RDRP technologies are: nitroxide mediated polymerization (NMP), atom transfer radical polymerization (ATRP) and reversible addition fragmentation chain transfer (RAFT) polymerization. All of these controlled polymerization mechanisms involve a polymerization mediator which in the case of RAFT polymerization helps to control the equilibrium between the dormant and active species during the propagation step [63,64]. Amongst these mechanisms, RAFT polymerization has shown to be the most powerful mechanism due to its versatility associated with mild reaction conditions, no use of transition metals, the possibility of using several types of monomers, including monomers with functional groups (e.g., OH, NR_2_, CO_2_H, SO_3_H, CONR_2_) and like others, allows well-controlled polymer architectures. Moreover, hydrophilic and amphiphilic polymers can be readily synthesized, which is important for bioapplications [64,65,66,67,68]. Additionally, RAFT polymerization has been successfully used for controlled functionalization of planar surfaces as well as micro- and nano-particles which is of major interest for a wide range of applications [1,68].

**Figure 5:**
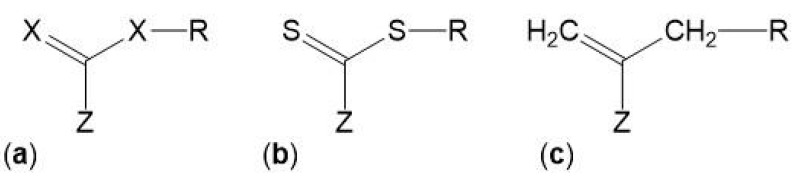
Structures of various chain transfer agent (CTAs)/RAFT agents.

The control over the composition and architecture of RAFT polymers depends on the kinetics of the reaction and the elimination/minimization of radical-radical terminations which is due to the use of a chain transfer agent (CTA), also known as RAFT agent. The CTA, with the structure shown in Figure 5, confers a “living” character to the polymerization since it mediates the polymerization via a reversible chain-transfer process and its efficiency depends on the nature of the groups X, Z and R (Figure 5a). Although CTAs where X = CH_2_ (Figure 5c) have been used, the most efficient CTAs are thiocarbonylthio compounds (Figure 5b) [65,66,69].

In turn, the Z and R groups are crucial for the efficiency of the addition-fragmentation reactions. The Z group controls the reactivity of the C=S double bond, influencing the rate of radical addition and fragmentation. The R group is the radical leaving group which also has to be able to reinitiate the polymerization for chain transfer. For an ideal RAFT agent, the dormant species should have a reactive C=S double bond, the intermediate radical should fragment rapidly and give no side reaction and lastly radicals (R•) should efficiently re-initiate polymerization [1,65,66,68,69,70,71].

**Figure 6:**
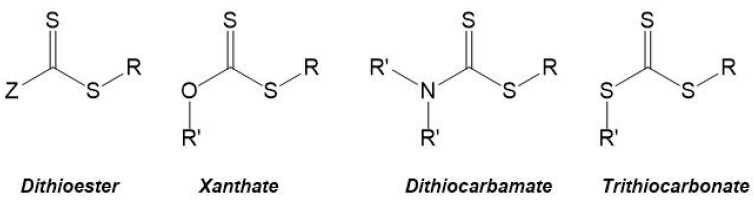
Generic structures of RAFT chain-transfer agents.

The most commonly used CTAs are the thiocarbonylthio compounds, with a general structure Z–(C=S)–S–R (e.g., dithioesters, xanthates, dithiocarbamates and trithiocarbonates—Figure 6), which have been used for the synthesis of different homopolymers and copolymers. Moad et al. 2005 and 2006 [65,66] summarized a wide range of these CTAs that were used until then, and Lowe et al. 2007 [70] focused on CTAs used in the synthesis of water-soluble copolymers.

The choice of the CTA is a key aspect for a successful RAFT polymerization and it depends not only on the properties of the R and Z groups but also on the monomer that will be polymerized, as well as the reactions conditions. Indeed, Moad et al. [65,66] have outlined some guidelines for the selection of RAFT agents based on the efficiency of the Z and R groups in the polymerization of some monomers such as methyl methacrylate (MMA), vinylacetate (Vac), styrene (S), methyl acrylate (MA), acrylamide (AM), and acrylonitrile (AN).

**Figure 7:**
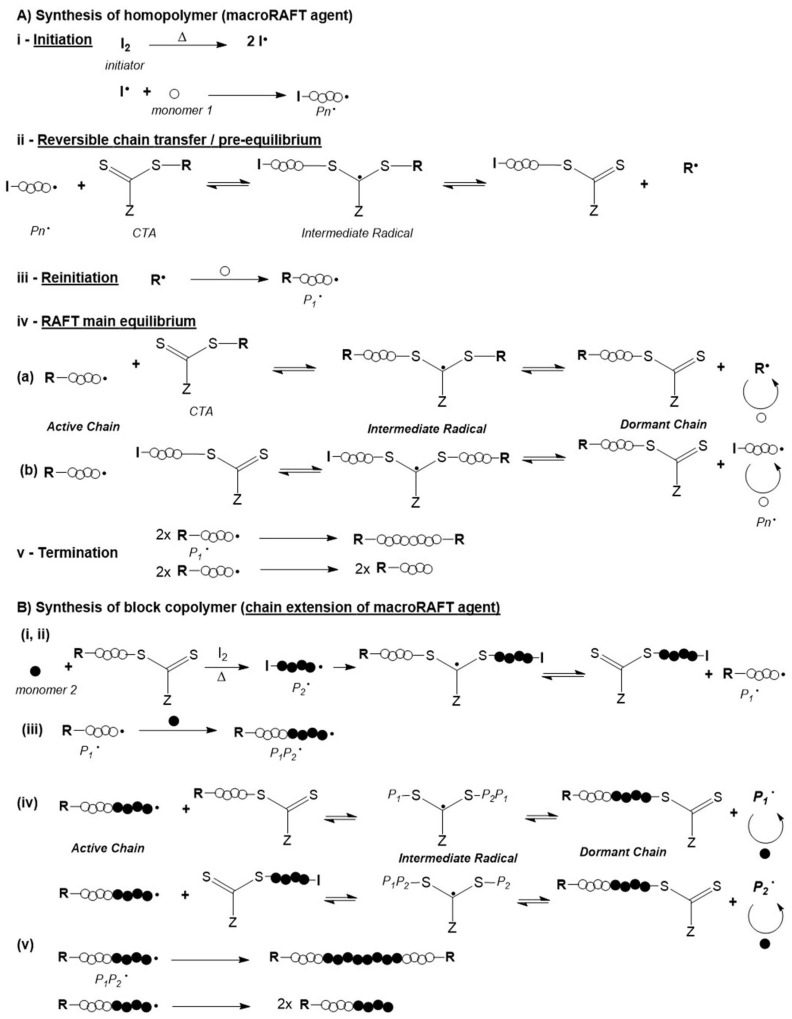
Proposed mechanism of RAFT polymerization; (I and II) homopolymerization and (III) chain extension of a macroCTA. Adapted from [67], Copyright 2008, with permission from Elsevier; and [73], Copyright 2009, with permission from John Wiley & Sons Inc. (Note that for the sake of clarity various dead polymer chains resulting from the different possible paths were omitted. Yet, they are discussed in the text above).

The RAFT polymerization mechanism is based on the reversible chain transfer and equilibrium between active and dormant chains, as illustrated in Figure 7A. It starts with the initiation step where a polymeric active chain (*P*n•) is formed via conventional radical polymerization. Then the primary radical formed reacts with the CTA, thus forming an intermediate radical. In turn, this intermediate radical suffers fragmentation resulting in a dormant polymeric chain (which contains the –S–C(=S)–Z of the CTA) and a radical species (*R*•). This radical species reacts with monomer (*M*) to form an active polymeric chain (*P*_1_•). The rapid equilibrium established between the active chain (i.e., the propagating radical) and the dormant chain (i.e., the polymeric chain which contains the CTA also referred to as macroCTA or macroRAFT agent) is fundamental to control the molecular weight, as well as to ensure that the concentration of dormant chains is greater than that of active chains [1,65,66,67,68,70].

The molecular weight of the polymer chains and its distribution, the composition and the architecture of the resulting polymers can be controlled due to the fact that the termination step is minimized. Suppression, or at least significant reduction of termination is due to the equilibrium that is established between the propagating polymeric chains and a macroCTA, also named macroRAFT agent. The CTA is preserved in the chain giving a “living” character to the macrochain which can be extended by adding a second monomer, as illustrated in Figure 7B. In fact, the resulting macroCTA can be isolated and subsequently used in another batch under distinct reaction conditions, for instance aqueous medium [65,66,67,68].

Essentially the control of molecular weight of the polymer chains is achieved by the ratio between the initial concentration of the monomer and the CTA ([*M*]_0_/[CTA]_0_), at a given initiator concentration. However, the full breast of molecular weight distribution (FMWD) can be rather complex. Even though the termination step is minimized it does occur and different reactions paths can compete as generally illustrated in a condensed manner in step (v) in Figure 7A,B respectively. Moreover, in the case of chain extension, the following types of dead polymers can occur: *R*-*P*_1_-*P*_1_-*R*, *R*-*P*_1_, *R*-*P*_1_-*P*_1_-*I*, *I*-*P*_1_, *I*-*P*_1_-*P*_1_-*I*, *R*-*P*_1_*P*_2_-*P*_2_*P*_1_-*R*, *R*-*P*_1_*P*_2_, *R*-*P*_1_*P*_2_-*P*_1_-*R*, *R*-*P*_1_*P*_2_-*P*_2_-*I*, *R*-*P*_1_-*P*_2_-*I*, *I*-*P*_2_, *I*-*P*_2_-*P*_2_-*I*, *I*-*P*_1_*P*_2_ and *R*-*P*_1_*P*_2_-*P*_2_*P*_1_-*I*. Furthermore, other types of termination involving intermediate adducts can also take place and different schools of thought have proposed different kinetic schemes and developed advanced calculation tools to simulate the FMWD and get a better understanding of the mechanism [72]. In fact, the kinetics of the various steps and mechanistic subtleties, associated with the type of RAFT agent, and reaction conditions are determinant to achieve tight control of the molecular weight distribution, where diffusional limitations can not be ignored. Nevertheless, broadening of MWD is often reported but it can be often due to poor handling of macroRAFT agents as the thiocarbonylthio groups is sensitive to oxidizing agents present in some solvents, as well as to pH, temperature and light [73]. Barner-Kowollik’s group, in particular, has been studying the kinetics and mechanisms of RAFT polymerization for a long time using a wide range of monomers, chain transfer agents and reaction conditions, including light initiation besides the standard thermal initiation. Studies carried out using pulse laser polymerization combined with size exclusion chromatography (PLS-SEC) and robust kinetic models have been frequently reported in this last decade. Recent examples of such studies include, for example the estimation of rate coefficients of backbiting reactions in acrylates polymerization [74] Another example consists in a comparative study of methods reported in the literature to experimentally determine RAFT transfer coefficients as well as a set of general guidelines for appropriate choice of the method to use [75]. More recently, the group reported the kinetic Monte Carlo model of PLS-SEC study on *n*-butyl acrylate which provided detailed information on chain propagation, chain initiation as well as termination reactivity [76]. These contributions consist in a major asset to develop models which can be used for in silico studies to screen reactants and polymerization conditions efficiently as opposed to conventional “try & error” approaches. Nevertheless, experimental validation is still required. Also worthy of notice is Keddie’s tutorial review which consists in a clear and didactic guide for the preparation of block copolymers via RAFT [77].

From what was outlined before, besides the relevance of the choice of the RAFT agent, experimental aspects should be also considered thus, examples of the effect of some polymerization conditions are briefly presented. For instance, RAFT polymerization started to be performed in organic medium being compatible with a wide range of organic solvents, but the solubility of the RAFT agent in the solvent should be a concern as well as the hydrolytic sensitivity of some RAFT agents to some solvents. Generally, RAFT polymerization in organic medium can be carried out over a temperature range from room temperature to 140 °C. Yet, whilst higher temperatures yield better results they can also promote conventional radical polymerization. Additionally, RAFT polymerizations can also be efficiently performed in aqueous medium thus enabling the polymerization of anionic, cationic, zwitterionic and neutral monomers leading to hydrophilic and functional polymers. In homogenous aqueous polymerization, the RAFT agents that have shown higher versatility are the dithioesters and trithiocarbonates. The use of RAFT polymerization in dispersed media has also been widely explored as it allows the preparation of nanostructures with a variety of morphologies, the combination of hydrophobic and hydrophilic monomers, as well as the preparation of stable colloids which can actually encapsulate inorganic nanoparticles as will be discussed later. Rieger has reported a rather comprehensive set of guidelines for the synthesis of block copolymer particles of distinct morphologies via RAFT polymerization conducted in dispersed media combined with stabilization through chain crosslinking using bifunctional monomers, which allows novel applications [78].

As regards the type of initiator used, any source of free radicals can be used such as azo and persulfate initiators, UV irradiation, γ-source, plasma field and thermal initiation in the case of styrene. Yet, studies have revealed that the choice of the initiator can also play a major role. For example Perrier et al. in their studies to optimize the preparation of multiblock copolymers via one-pot multistep sequential polymerization, used 2,2′-azobis[2-(2-imidazolin-2-yl)propane] dihydrochloride (VA-044) as initiator which has a rather short half-life at 44 °C (10 h) [79]. Furthermore, there are additional aspects related to the initiator that need to be taken into consideration: one is the concentration of initiator which is crucial to achieve a balance between the polymerization rate and the concentration of dead chains, usually the [CTA]_0_/[*I*]_0_ ratio is greater than one to ensure that there is a greater number of CTA molecules in solution than free radicals. Another aspect related to the initiator-derived radical is that it should be a good leaving group regarding the propagating radical [65,66,67,68]. The most used initiators are thermal initiators such as AIBN (2,2′-azoisobutyronitrile), ACPA (4,4′-azobis(4-cyanopentanoic acid)) and K_2_S_2_O_8_.

### 3.2. Application to Gold Nanocomposites

**Figure 8:**
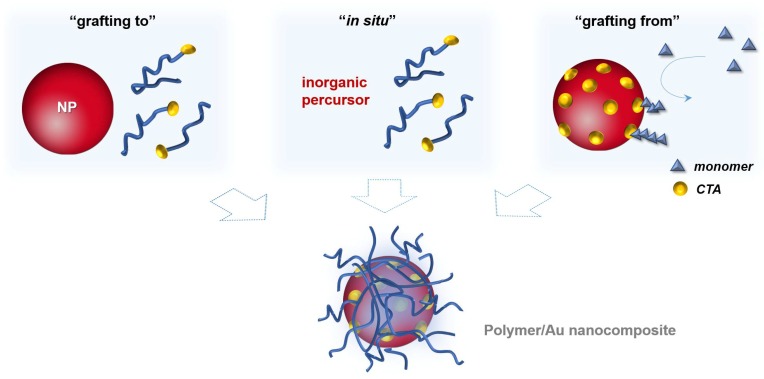
Common approaches to prepare polymer/Au nanocomposites.

In general, the surface modification of inorganic nanoparticles using RAFT polymerization has been explored following three main approaches that are schematized in Figure 8. In the “*grafting to*” and in the “in situ” strategies a previously prepared polymer is used. In the former, the polymer and the NPs are mixed and the polymer chains adsorb or bind covalently onto the NPs surface, respectively; whilst in the second strategy, NPs are synthesized using an inorganic precursor in the presence of the polymer. Regarding the third strategy, “*grafting from*”, the polymerization is carried out from the inorganic surface which is previously functionalized with the polymerization mediator (e.g., CTA) [1,68].

In the context of the preparation of polymer/Au nanocomposites, RAFT polymerization presents an advantage related with the use of CTAs. CTAs are usually a thiocarbonylthio compounds, therefore they have high affinity to gold surfaces due to the presence of sulfur atoms. Hence, polymers based on di-and tri-thio CTA agents have been used in the surface modification of gold nanoparticles due to the possibility of forming a strong linkage between the polymer and the NP surface [1,68].

**Figure 9:**
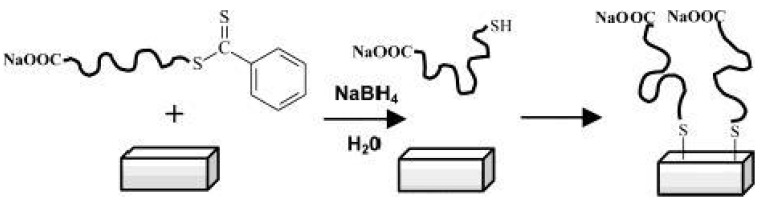
Mechanism describing the immobilization of a RAFT-prepared (co)polymer onto gold surface. Reprinted with permission from [80]. Copyright 2003 American Chemical Society.

Back in 2003 McCormick et al. [80] have reported the immobilization of polymers prepared by RAFT polymerization onto gold films. After the preparation of the polymers, poly(sodium 4-styrenesulfonate), poly((*ar*-vinylbenzyl)trimethylammoniumchloride), poly(*N*,*N*-dimethyl-acrylamide), and poly(3-[2-(*N*-methylacrylamido)-ethyldimethyl ammonio] propane sulfonate-*b*-*N*,*N*-dimethylacrylamide), the authors reduced the dithioester end group of the CTA to thiol using NaBH_4_. Then the thiol containing polymers were immobilized onto gold surfaces due to the high affinity of thiol to gold, as depicted in Figure 9.

However, in 2006 Fustin et al. [81] demonstrated that CTAs (dithioesters and trithiocarbonates) are also able to chemisorb onto gold surfaces, consequently their reduction into thiols is not mandatory. This demonstration was very helpful to increase the range of RAFT polymers that can be used for surface modification of gold, since some RAFT polymers are incompatible with NaBH_4_.

Regarding the three strategies schematized in Figure 8, all of them present advantages and disadvantages in the preparation of polymer/Au nanocomposites. The *grafting to* also referred as *post-modification* is the easiest and most straightforward methodology since each component is synthesized individually allowing control over the size and shape of the inorganic particle as well as the molecular weight, structure and composition of the polymer, and only afterwards are they mixed yielding the nanocomposite [1,68]. However, in this strategy, limited grafting density can be an issue, especially for polymers with high MW [82]. As mentioned before, chemisorption of the previously-prepared polymer onto Au NPs can be promoted through the thiol group, by removal of the RAFT agent [83,84,85,86,87] or can be carried out maintaining the RAFT agent (di- or tri-thio group). For example, hydrophilic polymers prepared by RAFT polymerization have been used to coat Au NPs. For instance, Davis et al. [88] 2010, reported the stabilization of previously prepared Au NPs (*d* = 20 nm) using temperature and pH responsive polymers prepared via RAFT polymerization: poly(2-aminoethylmethacrylamide) (PAEA), poly(acrylic acid) (PAA), poly(*N*,*N*-dimethylaminoethyl acrylate) (PDMAEA), poly(oligoethylene oxide) acrylate (P(OEG-A)), poly(oligoethylene oxide acrylate-*co*-diethylene oxide acrylate) (P(OEG-A-*co*-DEG-A)) and poly(*N*-isopropyl acrylamide) (PNiPAM). Only the cationic polymers required special care during mixing with Au NPs to avoid aggregation due to the interactions with the citrate anions at the Au NPs surface. Destarac et al. coated Au NPs (*d* = 8 nm) with three polymers prepared by MADIX/ RAFT polymerization which in aqueous solutions have different behaviors: a cationic polymer, poly[(3-acryl-amidopropyl) trimethylammonium chloride] (PAPTAC), a thermoresponsive polymer PNiPAM, and a pH-responsive polymer PAA [89], and also reported the used of poly(*N*-vinyl caprolactam) [55], which is also a thermoresponsive polymer. The optical properties of the resulting nanocomposites changed by varying the pH and/or temperature of the colloids depending on the polymer used. Klok et al. [90] in 2010 prepared Au NPs with different sizes from 5 to 47 nm which then were coated, in aqueous solution, with poly(poly(ethylene glycol)methacrylate) (*M*n = 16,800 g/mol) and demonstrated thermoresponsiveness. Klok et al. in 2011 [91] prepared a library of Au NPs (12, 28 and 51 nm) coated by post-polymerization modification of poly(pentafluorophenyl methacrylate) synthesized via RAFT polymerization with different chain lengths. This post-polymerization modification allowed the preparation of nanocomposites with different surface chemical functionalities (e.g., charge, polarity) and used them as optical sensors to study several biologically relevant media. In 2013, Vana et al. [92] coated Au NPs (*d* = 14 nm) with PNiPAM containing single or multiple trithiocarbonate (TTC) groups. The authors demonstrated that the TTC groups play an important role binding to the gold core, thus providing stability to the nanocomposite. Interestingly, using polymers with multiple TTC groups, the distance between the Au cores was kept constant even when polymers with higher MW were used. Vana’s group has also been working on the *grafting to* approach to prepare other controlled nanostructures with Au [93,94].

**Figure 10:**
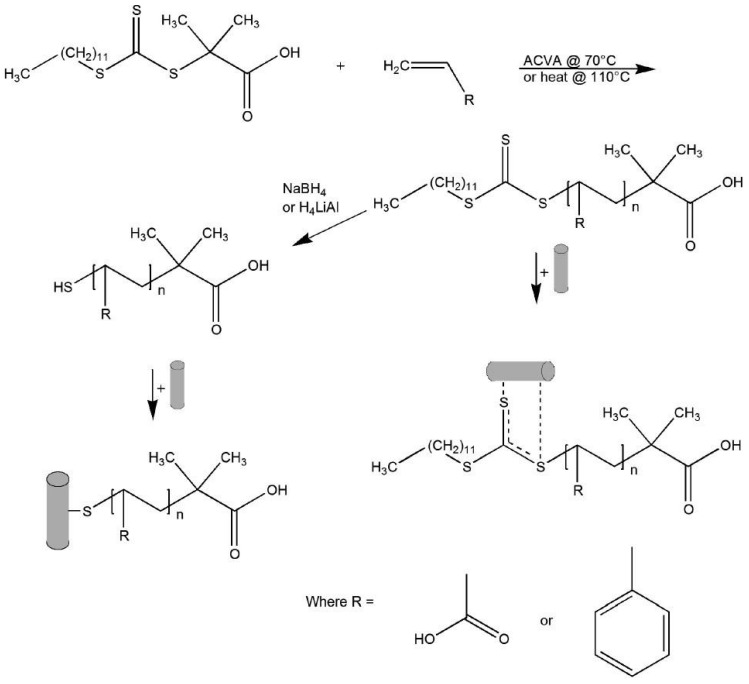
Proposed mechanisms describing synthesis, reduction, and immobilization onto a gold surface of RAFT-prepared poly(acrylic acid) (PAA) and polystyrene (PS). Reprinted with permission from [95]. Copyright 2007 American Chemical Society.

The surface of gold nanorods bearing polymers prepared via RAFT was reported by Boyes et al. in 2007 [95]. Gold nanorods (*AR* = 10) were firstly synthesized and subsequently modified with hydrophilic polymers poly(2-(dimethylamino)ethyl methacrylate) (PDMAEMA) or poly(acrylic acid) (PAA), in aqueous solution, and with the hydrophobic polymer (polystyrene—PS) in DMF. The authors demonstrated that the polymers were grafted to the Au nanorods surface by the di- or tri-thio group of the RAFT agent without the need to reduce these groups to the corresponding thiol. Figure 10 illustrates the synthetic procedure as well as the different coordination modes.

Destarac et al. [96] 2010 described the stabilization of previously prepared Au NPs (*d* = 8 nm) using not only a hydrophilic polymer (poly(*N*-isopropyl-acrylamide), PNiPAM) but also an amphiphilic diblock copolymer prepared by RAFT polymerization—poly(*n*-butyl acrylate-*b*-*N*-isopropyl-acrylamide) (PBA-*b*-PNiPAM). Interestingly, shell@core type nanostructures were obtained when the PNiPAM was used to stabilize the NPs, but when the PBA_2k_-*b*-PNiPAM_8k_ was used, free polymer globules were also obtained. Barros-Timmons et al. [97] have shown that using amphiphilic diblock polymer (P(PEGA_40_)-*b*-(MMA-*co*-BA)_140_-TTC) it is not possible to prepare, via this approach, core@shell type structures in aqueous medium. This happens because this amphiphilic copolymer formed well-defined micelles in water and the Au NPs, previously prepared, are not able to migrate to the core of the polymer sphere.

Actually, these two last examples demonstrate a disadvantage of the *post-modification* strategy in the preparation of polymer/Au nanocomposites towards bioapplications. The amphiphilic block copolymers in aqueous solutions can form well-organized aggregates, such as micelles, depending on their concentration in solution which compromises the adsorption of the polymer onto the Au surface. In fact, in what concerns the coating of Au NPs using the amphiphilic block copolymers, the methodology normally involves the use of organic solvents, such as THF or DMF, where organized aggregates are not formed [98,99,100].

**Figure 11:**
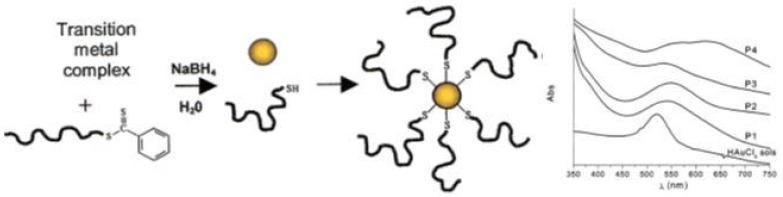
(**left**) Preparation of (co)polymer-stabilized transition metal nanoparticles, (**right**) UV-Vis spectra for HAuCl_4_ sols and polymer-stabilized Au NPs. P1—PAMPS, P2—PVBTAC, P3—PDMA, and P4—PMAEDAPS-*b*-PDMAm. Adapted with permission from [101]. Copyright 2002 American Chemical Society.

*In situ* preparation of this type of nanocomposites seems to be a very simple approach since it occurs in a one-pot procedure, i.e., the polymer with the desired molecular weight, composition and structure is prepared and the metal nanoparticles are generated in situ, usually using sodium borohydride (NaBH_4_) to reduce the inorganic precursor. However, this reducing agent can also reduce the dithioester- or trithiocarbonate-end groups of polymer chains leading to a thiol-ended polymer. This method involves several challenges such as the control over the size and shape of the nanoparticles [1,68]. Nevertheless, in 2002 McCormick et al. [101], demonstrated a novel route for the preparation of copolymer-stabilized gold nanoparticles via in situ preparation. The authors prepared four water soluble copolymers (anionic, cationic, neutral, and zwitterionic (betaine) species): poly(sodium 2-acrylamido-2-methyl propane sulfonate) (PAMPS), poly((*ar*-vinylbenzyl)-trimethylammonium chloride) (PVBTAC), poly(*N*,*N*-dimethyl-acrylamide) (PDMAm), and poly(3-[2-*N*-methylacrylamido]-ethyl dimethyl ammonio propane sulfonate-*block*-*N*,*N*-dimethylacrylamide) (PMAEDAPS-*b*-PDMAm). The metal salt (HAuCl_4_) was mixed with the dithioester end-capped copolymer and after addition of the reducing agent (NaBH_4_) copolymer-stabilized gold nanoparticles were obtained, as schematized in Figure 11. This procedure was also performed using the metal salts AgNO_3_, Na_2_PtCl_6_·6H_2_O and Na_3_RhCl_6_ resulting in the corresponding copolymer-stabilized nanoparticles. Whilst the authors proved that it is possible to synthesize stable metal nanoparticles in the presence of different RAFT polymers, studies focusing on the particle size control have not been performed.

In 2008, Kim et al. [102] prepared poly(ethylene oxide-*b*-*N*-isopropylacrylamide) by RAFT polymerization of NiPAM using PEO-based RAFT agent, varying the length of the PNiPAM block. The copolymer was mixed with HAuCl_4_, in THF, and reduced using NaBH_4_ dissolved in ethanol, yielding Au NPs with 5 to 30 nm in diameter regardless of the concentration and the length of PNiPAM block. Fan et al. [103] in 2011 reported the synthesis in situ of Au NPs in DMF using poly(styrene)-*b*-poly(ethylene oxide) (PS-*b*-PEO) block copolymer and a trithiocarbonate group located between the two blocks. The resulting Au NPs presented a diameter between 5 and 10 nm with λ_LSPR_ at 530 nm.

**Figure 12:**
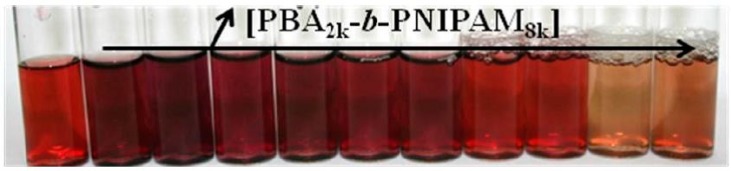
Image of the final NPs solutions 5 min after reduction at 20 °C. The polymer concentration increases from left to right (0 to 0.15 wt %). Reprinted with permission from [96]. Copyright 2010 American Chemical Society.

On the other hand, Destarac et al. [96] in 2010 reported the in situ synthesis of Au NPs, in water at pH = 8, using an amphiphilic diblock copolymer prepared by RAFT polymerization—poly(*n*-butyl acrylate-*b*-*N*-isopropyl-acrylamide) (PBA-*b*-PNIPAM). First, the authors observed that when a higher amount of polymer was used the reduction of the gold precursor was slower. Also, the color of the colloidal solution varied from dark brown to light orange depending on the polymer concentration, as shown in Figure 12. This is directly correlated with the size of the Au NPs generated: without polymer *d* = 6 ± 3 nm; for lower concentration (2.5 × 10^−3^ wt %) *d* = 8 ± 6 nm, and for higher concentration 1.5 wt % *d* = 1.6 ± 0.4 nm. It is noteworthy that the critical aggregation concentration of this copolymer is 3 × 10^−4^ wt %. Barros-Timmons et al. [97] have also shown that using amphiphilic diblock polymer (P(PEGA_40_)-*b*-(MMA*-co*-BA)_140_-TTC) the synthesis in situ of Au NPs in aqueous medium is influenced by the pH, the amount the copolymer, and the [HAuCl_4_]:[copolymer] ratio. Furthermore, in this case the Au NPs are generated in the outermost part of the polymer sphere (i.e., hydrophilic part).

In addition, in 2013 Marty et al. [104] demonstrated that the molecular weight of the polymer also influences the size of the Au NPs as well as the *end*-group of the polymer. In this study, the authors prepared PNIPAM by RAFT polymerization with three different molecular weights and then the terminal xanthate group was reduced to a thiol or a hydrogen. An aqueous solution of HAuCl_4_ was added to the polymer at pH = 8 and reduced with NaBH_4_. Indeed, as others had reported before, increasing the concentration of the polymer leads to a reduction of the NPs diameter. Additionally, small differences in the size were also found when different molecular weights were used. But significant differences in the size of the NPs, and consequently in the λ_LSPR_, were observed as function of the type of end-group (xanthate group—*X*, thiol—*SH*, and hydrogen—*H*). For instance, using higher PNIPAM concentration (0.1 wt %) diameters obtained are: *d*_H-end_ = 6.8 ± 3.0 nm, *d*_SH-end_ = 3.2 ± 0.4 nm, and *d_X-_*_end_ = 1.3 ± 0.8 nm; and the respective λ_LSPR_ are 545, 521 nm and no plasmon band was detected. These results clearly show that the presence of sulphur atoms has an important role during the generation of the gold nuclei and in the growth of the NPs. The authors noted that the xanthate group strongly binds to gold blocking the growth of the NPs. It is important to remember that the reducing agent (NaBH_4_) could also reduce the xanthate to thiol although the authors believe that this did not occur in their case.

In brief, despite of the apparent simplicity of the in situ synthesis, several parameters influence the generation of the NPs in the presence of the polymers. The molecular weight, the concentration and the chemical nature of the polymer influence the size of the NPs. Moreover, the hydrophilicity, hydrophobicity or amphiphilic nature of polymers and their subsequent configuration/organization in the solvent (aqueous or organic medium) also plays a significant role. However, the parameter that has the major impact is the functional groups containing sulfur, which strongly binds to gold and can block the growth of the NP. In general, for polymers containing sulfur, increasing the polymer concentration (consequently higher amount of sulfur atoms) results in a reduction of the size of NPs. Therefore, for each polymer, studies of the experimental conditions must be done in order to obtain the desired NP size in the nanocomposite.

*Grafting from* approach also known as surface-initiated controlled radical polymerization (SIP) is an elegant method which allows the well-controlled synthesis of the polymer from the surface of previously prepared nanoparticles. In this case, the nanoparticle is previously prepared, thus the size and the shape are controlled, being a methodology very attractive for coating anisotropic nanoparticles (e.g., nanorods). This methodology, requires two steps: (i) anchoring of the CTA onto the NPs surface and, (ii) the polymerization from NPs surface [1,68]. The former can be a challenge since the CTA grafting density could influence the second step, the polymerization, namely in what regards the chain length, and living characteristics of the polymer. Moreover, depending on the grafting taking place via the R group, or the Z group there are significant mechanistic differences. Stenzel has reported a rather systematic discussion regarding these two approaches, as well as the *grafting to* and in situ strategies and concluded that the *grafting from* is intrinsically more complex and difficult to control. Nevertheless, judicious choice of reaction conditions, including the use of sacrificial RAFT agents can yield good results [73].

Surface-initiated RAFT polymerization has been more widely explored using silica NPs, and has been reviewed by Böker et al. [105]. In the SIP, the CTA is covalently bonded to the silica surface and then the monomer is polymerized, in organic medium (e.g., DMF or THF) from the surface of the silica particle. Also using organic medium, back in 2003, Tenhu et al. [106] reported the RAFT polymerization of PNiPAM from a RAFT agent that was covalently grafted onto the surface of Au NPs (*d* = 3.2 nm) containing 11-mercapto-1-undecanol, in DMF. Barros-Timmons et al. have used this approach to prepare nanocomposites using CdS and CdSe quantum dots via miniemulsion [107]. Surprisingly, in aqueous media there are no reports regarding this type of Au NPs surface modification.

In the last years a similar strategy using a macroCTA or macroRAFT agent, instead of CTA has been explored. This approach, named RAFT assisted encapsulating emulsion polymerization (REEP), involves chain extension via RAFT emulsion copolymerization from the macroRAFT agent adsorbed onto NP surface. This approach was first reported in 2008 by Hawkett et al. [108] for the encapsulation of pigment NPs. The authors used a macroRAFT agent based on acrylic acid and butyl acrylate to stabilize the pigment and then a mixture of hydrophobic monomers (butyl acrylate and methyl methacrylate) was added in a controlled way. The macroRAFT agent acts as a surfactant during the RAFT emulsion polymerization thus allowing emulsion polymerization without the use of additional surfactants. Similar methodologies have been applied to several other particulates, such as platelets [109,110,111], carbon nanotubes [112,113], graphene oxide [114], cerium oxide (CeO_2_) NPs [115,116,117], and SiO_2_ NPs [111]. Nevertheless, in some methodologies additional surfactant was used, for example in the encapsulation of quantum dots (QDs) [118,119] and cerium oxide (CeO_2_) NPs [120]. More recently, this approach was reported to encapsulate Au NPs [97] without adding any additional surfactant, affording core@shell type structures. The authors adsorbed a macroRAFT agent based on poly(ethylene glycol)methyl ether acrylate onto Au NPs (diameter around 15 nm) and then the chain extension was promoted by adding a mixture of hydrophobic monomers (methyl methacrylate and *n*-butyl acrylate). Even though this approach is not yet well-explored to prepare polymer/Au nanocomposites, when the macroRAFT has a strong tendency to self-assemble in aqueous medium, this seems to be the most promising path to prepare core@shell type nanostructures in which the polymer shell can be designed, tailored and controlled [39]. However, this strategy is not as straightforward as it may seem. Indeed, the use of amphiphilic macroRAFT may in some cases be associated with self-assembly which prevents, or at least limits adsorption on the surface yet, very little is discussed in the literature regarding this aspect. Furthermore, adsorption of the macroRAFT onto the surface to obtain stable colloids (which is crucial for successful REEP) has proven rather complex as it depends on the chemical and structural characteristics of the nanoparticles surface as well as of the chemical and structural characteristics of the macroRAFT used. Bourgeat-Lami et al. and Barros-Timmons et al. have reported studies which highlight these issues [121,122].

## 4. Au NPs in Opto-Biodetection and Beyond

The leading interest of Au NPs in bioapplications, namely in biosensing applications, is mainly due to the existence of LSPR, which is very sensitive to variation of the surrounding environment, including the state of aggregation of the NPs and their behavior in the presence of fluorophores. More specifically, these features allow the use of gold NPs in systems in order to identify specific (bio)analytes that induce the aggregation of NPs or, induce changes in the fluorescence (e.g., quenching effect) of a fluorophore close to the surface of the Au NPs. For example, these systems can be used in the identification of a specific strand of DNA, antibody-antigen or proteins-ligand [3,123,124,125,126,127].

**Figure 13:**
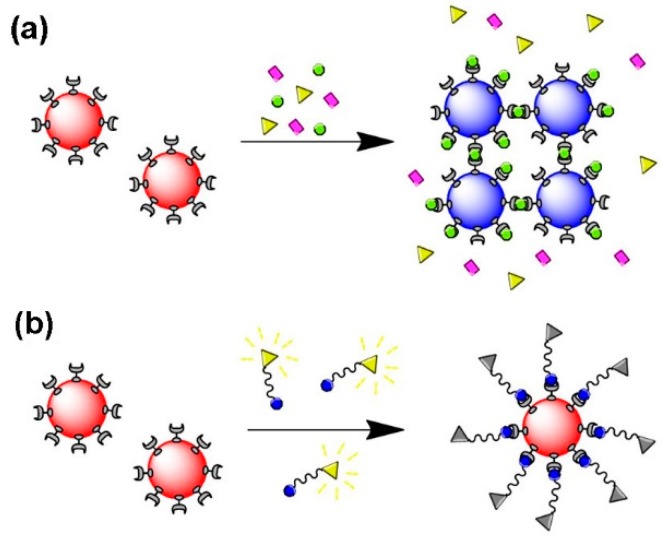
Schematic representation of Au NPs biosensor based on LSPR (**a**) and based on quenching fluorescence (**b**).

Several biosensors envisage the identification of biomolecules, such DNA and proteins, that result from aggregation of Au NPs induced by specific molecular recognition, thus shifting the LSPR band, a phenomenon that in certain cases can be observed by visual inspection. Hence, colloidal Au NPs that are well dispersed in water present a characteristic color, but when aggregated induce color changes, typically from red to purple-blue, depending on the particle aggregation state [58,128,129,130,131] Figure 13a shows a schematic representation of Au NPs as a biosensing platform based on the LSPR. Regarding biosensing based on fluorescence quenching, receptor/ligand binding and release events can be monitor through changes in fluorescence intensity or lifetime of the fluorophore, reflecting binding and unbinding states of analytes to sensors, since the fluorescence intensity is dependent on the distance between the Au NP (quencher) and the fluorophore [16,125,131,132,133,134,135,136] (Figure 13b).

The development of optical biosensors based on RAFT-polymer/Au nanostructures have been less exploited. Examples include the report of a biorecognition event (bioreceptor/bioanalyte) for the system sugar/lectin (e.g., glucose or mannose/Concanavalin A). For instance, glucose containing monomers are polymerized in an aqueous solution via RAFT polymerization and after reducing the dithio group from the Z-terminal to a thiol termination, the thiolated glycopolymer is mixed with Au NPs via a “*grafting to*” approach. The biorecognition event is observed by the shift of the LSPR band to longer wavelengths and decrease of absorbance, which is the result of aggregation due to the increase of the concentration of Concanavalin A [137]. In a similar work, instead of glucose, the authors have used mannose moieties which showed stronger biorecognition abilities by increasing the density of this bioreceptor in the polymer shell [86]. In another example, the authors have prepared via RAFT polymerization a polymer containing *N*-(2-hydroxypropyl)methacrylamide (HMPA) and *N*-(3-aminopropyl)methacylamide (APMA). While the HMPA is known by its non-immunogenic nature, the APMA is used to provide amine groups where a trisaccharide was covalently attached. The UV-Vis spectrum of glycopolymer-SH mixed with Au NPs showed a red-shift in the LSPR and a decrease in absorbance, and macroscopic aggregation of these functional NPs was even observed in the presence of the specific lectin. Furthermore, transmission electron microcopy analysis has shown that these multivalent nanostructures have specific affinity to hemagglutinin, which are proteins present on the surface of the influenza virus; nanostructures with no affinity to hemagglutinin, and used as control systems, were not observed around the surface of the virus [138]. Interestingly, other glycopolymer-stabilized Au NPs prepared by a similar strategy as mentioned above, were explored as platforms for synthetic anticancer vaccines [139]. In addition, Gibson et al. [82] coated Au NPs with poly(oligoethyleneglycol methacrylates) (POEGMA) and poly(*N*-vinylpyrrolidones) (PVP) with different MW prepared via RAFT, and compared the grafting density, stability in buffer solutions, temperature sensitivity and the optical response to a lectin. The Au nanostructures based on PVP and with lower MW presented higher grafting density. Note that the grafting density is an important parameter in cases where the biofunctionality (e.g., sugar moiety) is at the end of the polymer chain, thus graft density is proportional to the number of biorecognition moieties. The authors even linked amino-glicosides to the carboxylic acid group at the chain terminal, and observed that the shorter PVP@Au nanostructures showed stronger binding of Con A.

In the area of diagnosis, Au NPs can also be used as surface enhanced Raman scattering (SERS) platforms. This effect also results from the interaction of the electromagnetic radiation with the Au NPs, resulting in the local enhancement of the electric field in specific sites (hot spots), such as metal nanojunctions. Thus, the polarization of molecules adsorbed at the surface of the NP are also affected resulting in an enhancement of the Raman scattering signal, allowing the identification of such molecular species even in very diluted conditions [140,141,142]. The importance of polymer/Au nanocomposites in the preparation of these diagnosis platforms also results from the colloidal stabilization and chemical functionalization conferred by the polymer shells, namely when they are intended to be used in complex environments such as real biological samples [141,142,143] RAFT-polymer/Au nanocomposites have been prepared aiming the preparation of nanoassemblies, in which the NPs become close to each other, thus creating “hot spots” that result in improved SERS signals [144].

Additionally, the polymer shell in the plasmonic platforms composed by polymer/Au nanocomposites, can also provide responsive behavior by a judicious choice of the monomers. For instance, polymers based on poly(ethylene glycol) (PEG) or poly(ethylene oxide) (PEO), poly(*N*-isopropyl acrylamide) (PNIPAM) and poly(*N*-vinylcaprolactam (PNVCL) provide thermal responsiveness and acrylic acid pH responsiveness, as will be discussed below. Moreover, polymers based on PEG/PEO can also afford biocompatibility and antifouling properties to the nanocomposites, which is relevant to limit non-specific interactions between surfaces and proteins. These “smart” nanomaterials can be used in drug/gene delivery applications and the role of RAFT polymerization is very important here, because it allows control the molecular weight and composition of the polymer shell, which is crucial to tailor and adjust the sensitivity of the polymer to an external stimulus. 

In many works reporting RAFT-polymer/Au nanocomposites, only the concept of shell response to an external stimulus (such as change of pH and/or temperature) is demonstrated but without real applications for these “smart” nanomaterials. In the RAFT polymerization of NiPAM, using a PEO-based xanthate-type RAFT agent, the authors have demonstrated the influence of the PNiPAM molecular weight (MW) on the low critical solution temperature (LCST). Then in the presence of this polymer, Au NPs were synthesized using sodium borohydride as reducing agent (in THF/ethanol) and the resulting nanocomposite proved to be stable in water, however increasing the temperature the transparent colloid becomes turbid [102]. Destarac et al. have shown the impact of the MW balance of two blocks (PNiPAM and butyl acrylate, BA) on the cloud point value of the block copolymer. Here, the preparation of polymer/Au nanocomposites was performed via both *grafting to* and in situ strategies; in this case, reversible changes occur in LSPR by increasing the temperature, as a result of polymer shell conformation [96]. Moreover, these authors have demonstrated that by increasing the NaCl concentration, the cloud point of the block copolymer decreased significantly. The same behavior was observed later for PNiPAM@AuNPs also prepared via RAFT polymerization. Unlike the nanocomposites dispersed in pure water, using a NaCl aqueous solution, the optical properties of the colloid are more sensitive to the increase of temperature leading to a red-shift and absorbance decrease in the LSPR band. This effect is the combination of ionic screening of citrate ions at the NPs surfaces and a salting out effect on PNiPAM chains, which lead to dehydration and consequently an increase of hydrophobic interactions [84,145]. Furthermore, it was found that free PNiPAM in solution promotes the aggregation of PNiPAM/Au nanostructures when the temperature increases, because it works as a cross-linking agent [146,147]. More recently, Gibson et al. have shown a co-operative aggregation behavior using PNiPAM@Au NPs with different cloud point. This parameter was tuned by controlling the MW of PNiPAM chains and the Au NP size [148]. In another thermoresponsive Au nanocomposite based on PNiPAM, with different MW, a fluorophore was used in order to prepare a biosensing platform based on fluorescence quenching to detect six distinct proteins. Depending on the nature of the proteins (MW and isoelectric point—pI), different interactions with the nanostructures were observed by monitoring the photoluminescence emission spectra [83].

PEG-based polymers are also known to have thermosensitivity, and Davis et al. [149] have shown that the LCST can be tuned (from 15 to 90 °C) by adjusting the ratio of the monomers, oligoethylene glycol acrylate (OEG-A) and di(ethylene glycol) ethyl ether acrylate (DEG-A), during the RAFT polymerization. Likewise, the poly(OEG-A-*co*-DEG-A)/Au NPs have shown thermoresponsiveness but also protein antifouling properties. Later, a similar study, was performed by using the methacrylate group in these monomers, because the hydrophilicity of the backbone is slightly different and has influence in the LCST. The authors have explored the impact of using statistical or diblock copolymers in the thermal response [87,150]. Moreover, homo and statistical copolymers prepared by RAFT polymerization, using *N*-vinylpyrrolidone and *N*-vinylcaprolactam, were grafted to Au NPs and presented temperature-responsive behavior in which the sensitivity increases with NaCl, in the range from 25 to 60 °C [151]. Other authors go further by producing polymer/Au nanocomposites with dual properties, pH- and temperature-sensitivity. For example, Au NPs were generated in the presence of poly(2-dimethylaminoethyl methacrylate) three-arm star polymers and the resulting hybrid nanostructure showed different LCST values (35 and 45 °C) for pH 10.5 and 8.4, respectively, as demonstrated by dynamic light scattering (DLS) measurements. Both pH values are above the corresponding p*K*_a_, where hydrophobic interactions take place with an increase of temperature [152]. Also using polymers based on NiPAM, the pH and temperature responsiveness were explored for PNiPAM/Au nanocomposites at pH below the p*K*_a_. In these conditions, by increasing the temperature above the LCST, the nanocomposites migrate to an organic phase (chloroform), due to the increase of hydrophobic interactions, and this behavior was shown to be reversible [153].

More recently, some works have reported applications of RAFT-polymer/Au nanostructures which are sensitive to pH and/or temperature and that are not limited to biodetection. As already mentioned, the “smart” nanomaterials cited above are promising platforms to be used in drug delivery systems for example in cancer therapy. Because cancer cells exhibit a microenvironment where the pH is slightly acidic, this can trigger the release of the drug by using a pH-sensitive polymer. On the other hand, by using thermal sensitive polymers, the drug release can be promoted by local heating [154,155]. For example, Becer et al. [153] polymerized methacrylic acid (MAA) via RAFT polymerization and after reducing the RAFT end group, the thiolated PMAA was grafted to the Au NPs surfaces (*d* ~ 50 nm). Subsequently, doxorubicin (DOX), which was employed as a model anticancer drug, was linked using cysteine (Cys) and a crosslinker. This latter linkage, hydrazone linkage, is sensitive to pH. First, drug release experiments were carried out in PBS at pH values of 7.4 and 5.3, simulating the healthy and cancerous cellular environments, respectively, and then in cell cultures. These Au NP-PMAA-Cys-DOX nanostructures seem to be very promising platforms for theranostic treatments, combining chemotherapy/radiotherapy and fluorescence imaging. A similar work was performed by the same group but here the authors synthesized by RAFT polymerization, not only MAA but also OEGMA and/or mannose-containing monomer. The incorporation of these carbohydrates allows the guiding of the nanocomposites to the cancer cells, via biorecognition processes, enhancing the target exposure to the drug [156].

The thermoresponsiveness of certain polymers can be combined with the photothermal behavior of Au NPs in phototherapy, eventually combined with chemotherapy. These plasmonic photothermal properties result from light excitation matching the LSPR that leads to local heating, thus can be used to increase the local temperature in tumors, provoking protein denaturation (>42 °C) and consequently death of the cancer cells. Moreover these metal NPs are less invasive than conventional photoabsorbing dyes because they present a higher absorption cross section, so are more effective using lower excitation energies. In the specific case of Au NRs, the shift of the longitudinal mode to the therapeutic NIR window provides an optimal region to use for application in tissues and aqueous environments, due to minimal absorption. Thus Au NRs and plasmonic nanostructuctures with a LSPR band in the NIR region (e.g., nanoshells) are excellent candidates for photothermal therapy [157]. For instance, the copolymer poly(ethylene glycol)_114_-*b*-poly(*N*-vinylcaprolactam)_237_ (PEG-*b*-PNVCL) with a LCST at 39 °C was synthesized via RAFT polymerization and then grafted onto Au NRds surface. The GNR@PEG-*b*-PNVCL was loaded with rhodamine B, as hydrophilic drug model, and drug release tests were performed by using a NIR laser (λ = 802 nm). These smart drug delivery systems have shown biocompatibility and are promising nanomedicines for chemotherapeutic and phototherapeutic functions [158]. All these in vivo applications must address human safety issues, biocompatibility and cytotoxicity. There are several works that show that these “smart” nanomaterials are promising to be used in biomedicine [155,158,159,160,161].

## 5. Conclusions

This review highlighted the relevance of surface modification and functionalization methods in achieving functional Au NPs for opto-biodetection methods. Although a large number of publications have reported different approaches aiming at these type of core-shell nanostructures (e.g., small charged molecules, polyelectrolytes or thiolated molecules), polymers offer convenient routes to prepare stable, robust and multifunctional shells while keeping the optical properties of the Au core. In particular, RAFT polymerization has emerged as a powerful tool to obtain coated Au NPs with controlled polymer shells. Furthermore, RAFT polymerization can use functional monomers and mild reactions conditions which is a great advantage of this controlled radical polymerization mechanism. Three strategies were presented and discussed in this review: “grafting to” involves the mixture of previously prepared polymers with Au NPs and quasi-covalent interactions such as sulfur-Au, are involved. In this case, full control over the polymer (MW) and the NP (size and shape) are advantageous but polymer graft density can be a challenging issue especially in cases involving small NPs (<10 nm) or polymers with high MW. In the “in situ” strategy, NPs are synthesized using an inorganic precursor in the presence of the previously prepared polymer. This one-step nanocomposite synthesis is very interesting however achieving control over the size of the NPs (usually nanospheres) is not straightforward since it depends on many factors, namely the chemical functional groups and the MW of the polymers. As regards the “*grafting from*” approach, the Au NPs are previously prepared with the desired optical properties by using size and shape as control parameters. The polymer shells are then prepared and tailored from the NP surface modified with RAFT agents, keeping the control over the MW, although smaller MW are usually obtained, higher grafting densities are also achieved. Even though this is a very promising strategy, the polymerization mechanism can be rather complex thus it requires fine tuning of various reaction parameters. In the last years, a similar strategy (REEP) has been developed for the design of more robust polymer shells. REEP involves chain extension via RAFT emulsion copolymerization from the macroRAFT agent adsorbed onto NP surface. All these strategies are potentially useful for biomedical applications but still present challenges, that include issues regarding toxicity and biosafety assessment.
